# Appropriateness of hospitalization for CAP-affected pediatric patients: report from a Southern Italy General Hospital

**DOI:** 10.1186/1824-7288-35-26

**Published:** 2009-09-02

**Authors:** Fabio Antonelli, Daniele De Brasi, Paolo Siani

**Affiliations:** 1 Pediatric Unit, AORN "A. Cardarelli", Naples, Italy

## Abstract

**Background:**

Community-acquired pneumonia (CAP) is a common disease, responsible for significant healthcare expenditures, mostly because of hospitalization. Many practice guidelines on CAP have been developed, including admission criteria, but a few on appropriate hospitalization in children. The aim of this study was to evaluate appropriate hospital admission for CAP in a pediatric population.

**Methods:**

We evaluated appropriate admission to a Pediatric Unit performing a retrospective analysis on CAP admitted pediatric patients from a Southern Italy area. Diagnosis was made based on clinical and radiological signs. Appropriate hospital admission was evaluated following clinical and non-clinical international criteria. Family ability to care children was assessed by evaluating social deprivation status.

**Results:**

In 2 winter seasons 120 pediatric patients aged 1-129 months were admitted because of CAP. Median age was 28.7 months. Raised body temperature was scored in 68.3% of patients, cough was present in 100% of cases, and abdominal pain was rarely evidenced. Inflammatory indices (ESR and CRP) were found elevated in 33.3% of cases. Anti-Mycoplasma pneumoniae antibodies were found positive in 20.4%. Trans-cutaneous (TC) SaO_2 _was found lower than 92% in 14.6%. Dyspnoea was present in 43.3%. Dehydration requiring i.v. fluid supplementation was scored in 13.3%. Evaluation of familial ability to care their children revealed that 76% of families (derived from socially depressed areas) were "at social risk", thus not able to appropriately care their children. Furthermore, analysis of CAP patients revealed that "at social risk" people accessed E.D. and were hospitalized more frequently than "not at risk" patients (odds ratio = 3.59, 95% CI: 1,15 to 11,12; p = 0.01), and that admitted "at social risk" people presented without clinical signs of severity (namely dyspnoea, and/or SaO2 ≤ 92%, and/or dehydration) more frequently than "not at risk" population (p = 0.005).

**Conclusion:**

Dyspnoea was found to be the main clinical criterion to define an appropriate children admission for CAP. Other more objective evaluation (i.e. oxygen pulse oxymetry) could underestimate the necessity of hospitalization as patients discomfort could be more severe then indicated by TC SaO2. Furthermore, family inability to children care represents the main criterion for hospital admission in our geographic area. It reflects social deprivation status and it should be strongly considered in deciding for children hospital admission.

## Background

Community-acquired pneumonia (CAP) is a common disease and care of CAP is responsible for significant healthcare expenditures, most of which are represented by hospitalization of patients [1]. Practice guidelines and critical pathways have been developed to optimize the care of patients with CAP, particularly concerning hospital admission criteria. In pediatric population more specific recommendations on assessing clinical severity have been established, and admission criteria include both clinical and social parameters [2]. A lot of systematic evaluation on hospital admission appropriateness for CAP were performed in adult population [3,4], but this issue has been rarely estimated in children [5]. In the present report we assessed appropriate admission to a Pediatric Unit because of CAP.

## Methods

To assess appropriate admission to a Pediatric Unit of a Main General Hospital of Southern Italy (namely Pediatric Unit of AORN "A. Cardarelli" of Naples), we performed a retrospective analysis on admitted pediatric patients affected by CAP. Patients were from Naples and surrounding areas. Diagnosis was made based on clinical and radiological signs of CAP in all patients [2]. Patients were divided in 3 groups: infants (1-12 months), preschool children (13-60 months), and school children (61-129). Infants with clinical signs of respiratory syncytial virus infection were not included in the study. Raised body temperature (> 38°C), cough, and abdominal pain were also evaluated. As to laboratory findings, erythrosedimentation rate (ESR), C-reactive protein (CRP), and anti-Chlamydia pneumoniae and anti-Mycoplasma pneumoniae antibodies were assessed and considered positive according to laboratory cut-off. Appropriate hospital admission was evaluated following international criteria [2,6] as follows: for infants, oxygen saturation (SaO_2_) ≤ 92% and/or cyanosis, dyspnoea, intermittent apnea/grunting, not feeding, ability of family to provide appropriate observation or supervision; for older children: oxygen saturation ≤ 92% and/or cyanosis, dyspnoea, grunting, dehydration, ability of family to provide appropriate observation or supervision. Dyspnoea was defined by the presence of at least one of the following parameters: raised respiratory rate (compared with age specific normal ranges), chest retractions, and nasal flaring [6].

Family ability to care children was assessed by evaluating the social deprivation status related to living areas of patients, as indicated by school graduation area mapping [7,8]. In detail, a "deprivation score" was generated based on education level, in respect to the geographic area of origin of people. Naples city and surrounding areas were analyzed [8]. Prevalence of low school graduation people (evaluating primary school graduation, no school graduation literate, and illiterate people) of the single geographic area was considered the specific "area score" (data not shown). The higher was the score, the higher was the social risk. The "deprivation score" was generated arbitrarily: single area score was compared with national (Italian) score (0.36), considered "risk cut-off": areas with a lower score (≤ 0.36) were considered "not at social risk"; areas with a higher score (≥ 0.37) were considered "at social risk". If people were from areas with a score > +2SD than national score (namely ≥ 0.45), they were considered "at high social risk" (see Table 1).

**Table 1:** Deprivation score generated by evaluating educational level of people from Naples and surrounding areas (for details see the text).

	**Deprivation score**		
Group	Not at social risk	At social risk	At high social risk

Score	≤ 0.36	≥ 0.37	≥ 0.45

Children with pre-existing respiratory diseases and patients requiring intensive respiratory care after admission were not included in the study. Statistics was performed using the StatsDirect Statistical software version 2.7.5 (StatsDirect Ltd, Altrincham, UK). For comparison of hospital admissions and presence of clinical admission criteria between "at social risk" and "not at social risk" population, we estimated Odds ratio (OR) and used χ^2 ^test for univariate analysis of data, based on 2 × 2 cross-tabulations. Significance was defined as p < 0.05.

## Results

On a total of 11,080 pediatric patients examined at E.D. in winter seasons 2006-2007 and 2007-2008, 2,770 were admitted to our Hospital Unit (25%). In the same period, 135 CAP (radiologically confirmed) affected patients were examined at E.D.: 15 children were not admitted, 120 were admitted (88.8%); incidence of CAP on total admission was 4% (120/2,770). Non-admitted patients presented with normal clinical parameters; among them deprivation score was under the cut-off in 8/15 (53.3%), above the cut-off in 7/15 (46.6%), whose 5 "at high social risk" (33.3%).

Age range of admitted patients was 1-129 months with median age of 28.7 months, infants representing 37.5% (45/120), preschool children 50.8% (61/120), and school children 11.6% (14/120) (see Table 2). Sex was equally distributed (61 males/59 females). Admissions were slightly prevalent during week-end: 20% on Saturday, Sunday and holydays vs. 15% on other days of the week (average).

**Table 2:** Demographic and clinical characteristics of children with CAP.

**Characteristic of patients**	
Total patients observed at E.D.	135

Total admitted patients	120

Age (months)	28.7 (range 1-129)

Infant	45/120 (37.5%)

Preschool children (13-60 months)	61/120 (50.8%)

School children (60-129 months)	14/120 (11.6%)

Sex	

Male	61 (50.8%)

Female	59 (49.2%)

Fever	82/120 (68.3%)

Cough	120/120 (100%)

Abdominal pain	4/120 (3.3%)

Pneumonia diagnosed at chest roentgenogram	120/120 (100%)

Elevated inflammatory indices (ESR, CRP)	40/120 (33.3%)

Positive anti-Mycoplasma pn. antibodies	17/83 (20.4%)

Positive anti-Chlamydia pn. antibodies	0/83

ESR, erythrosedimentation rate; CRP, C-reactive protein.

Seven children admitted because of CAP were from developing countries: 6 were from "at social risk" areas (4 from "at high social risk"); a single patient was from a "not at social risk" area. No immigrant was scored among non-admitted patients.

Raised body temperature was scored in 82/120 patients (68.3%), 17/45 infants (37.7%), 51/61 preschool children (83.6%), and 14/14 school children (100%). Cough was present in 100% of cases. Abdominal pain was rarely evidenced: 3.3% of total patients (4/120); none among infants; 4.9% among preschool children (3/61); and 7.1% among school children (1/14).

Inflammatory indices (ESR and CRP) were found elevated in 40/120 (33.3%) patients. ESR was found elevated in 6/45 (13.3%) among infants, 30/61 (49.1%) among preschool children, and 5/14 (35.7%) among school children. CRP was found raised in 6/45 (13.3%) among infants, 27/61 (44.2%) among preschool children, and 8/14 (57.1%) among school children. Combination of ESR and CRP evaluation revealed that at least a single parameter was found raised in 10/45 (22.2%) among infants, 36/61 (59%) among preschool children, and 9/14 (64.2%) among school children. Anti-Mycoplasma pneumoniae antibodies were performed in 83 patients/120, regardless of age. Positivity was scored in 17/83 (20.4%), in particular in 1/45 (2.2%) among infants, in 14/61 (22.9%) among preschool, and in 3/14 (21.4%) among school children. Anti-Chlamydia pneumoniae antibodies were not scored in any tested patient. Summary of general and clinical characteristics of patients is reported in Table 2.

SaO_2 _evaluated in all patients was found lower than 92% in 18 (15%), requiring oxygen supplementation. Infants with SaO_2 _≤ 92% were 7 out of 45 (15.5%). Preschool children presented low SaO_2 _in 10/61 (16.3%); a single school child with low SaO_2 _was scored (7%).

Dyspnoea was present in 52/120 (43.3%) patients; percentage of dyspnoeic patients was higher among infants (28/45, 62.2%) than older ages (31.1% and 21.4% for preschool and school children, respectively). All patients presenting with low SaO_2 _also had dyspnoea.

Dehydration requiring i.v. fluid supplementation was scored in 16/120 patients (13.3%), 7/45 among infants (15.5%), 7/61 among preschool (11.4%), and 2/14 among school children (14.2%). Intermittent apnea or grunting was not scored.

Assessment of familial ability to children care revealed that 91/120 (76%) families derived from socially depressed areas (as resulted from the analysis of geographic areas), considered not able to provide appropriate child observation or supervision. Among these, 30/91 (33%) families were at high risk of social deprivation status. Among infants, 35/45 (78%) were at social risk, 24% of cases "at high social risk". Among preschool children 47/61 (77%) resulted "at social risk", and 9/14 (64%) among school children.

Evaluation of admission criteria for hospitalized children and infants with CAP is summarized in Table 3.

**Table 3:** Evaluation of hospital admission criteria for total hospitalized children with CAP, infants, preschool children, and school children.

**Criteria for hospital admission**	**Number of patients/total (%)**			
	
	**Infants**	**Preschool**	**School**	**Total**
Oxygen saturation ≤ 92% or cyanosis	7/45 (15.5%)	10/61 (16.3%)	1/14 (7.1%)	18/120 (15%)

Dyspnoea	28/45 (62.2%)	20/61(31.1%)	3/14(21.4%)	52/120 (43.3%)

Not feeding/dehydration	7/45 (15.5%)	7/61(11.4%)	2/14(14.2%)	16/120 (13.3%)

Inability of family to child supervision	35/45 (78%)	47/61 (77%)	9/14 (64%)	91/120 (76%)

Intermittent apnea/grunting	0/45	0/61	0/14	0/120

**Figure 1:**
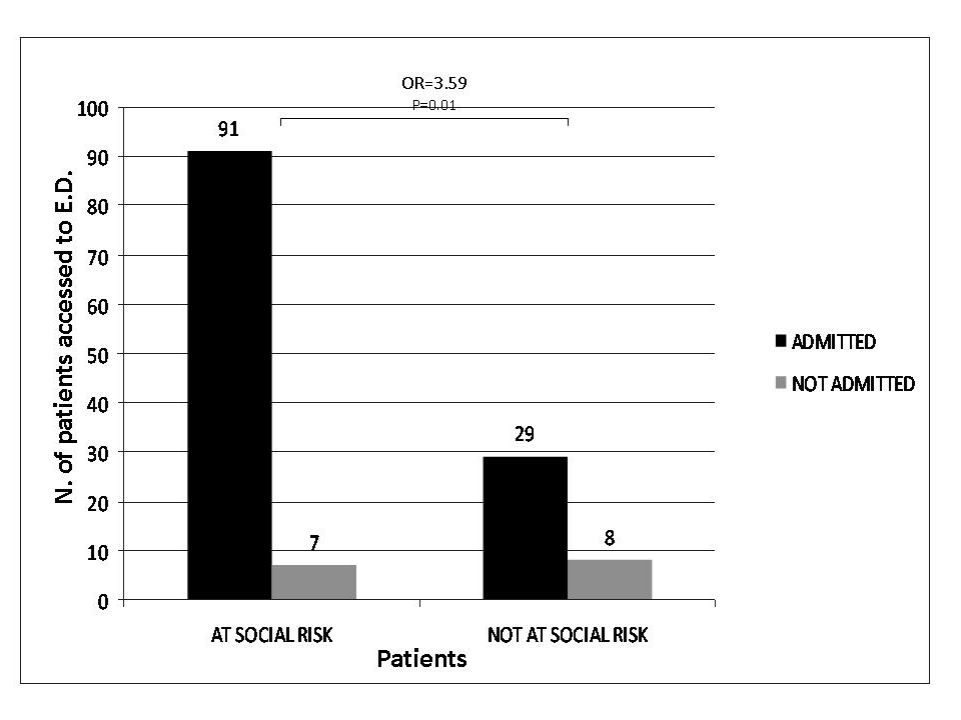
**Evaluation of Hospital admission/not admission in "at social risk" vs. "not at social risk" patients: "at social risk" patients were admitted more frequently than "not at social risk" patients (OR = 3.59, 95% CI: 1,15 to 11,12; p = 0.01)**.

**Figure 2:**
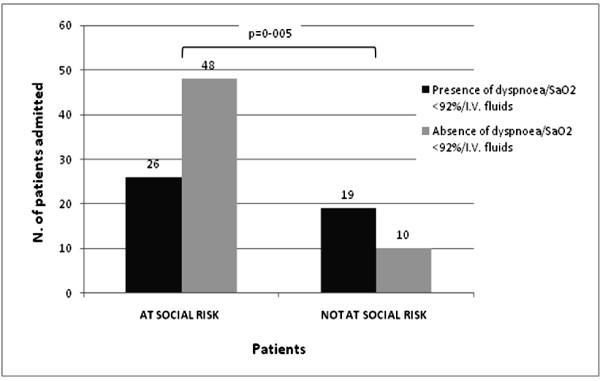
**Comparison of combined clinical admission criteria (i.e. dyspnoea, SaO2 ≤ 92%, i.v. fluids administration) among admitted "at social risk" patients vs. admitted "not at social risk" patients (p = 0.005)**.

Analysis of CAP patients revealed that "at social risk" people accessed E.D. and were hospitalized more frequently than "not at risk" patients (OR = 3.59, 95% confidence interval: 1,15 to 11,12 p = 0.01; see Figure 1). Furthermore, among admitted "at social risk" patients, combination of dyspnoea, and/or SaO2 ≤ 92%, and/or dehydration was scored in 26/74 cases (35%); in contrast, 19/29 admitted patients with no social risk (66%) presented with dyspnoea, and/or SaO2 ≤ 92%, and/or dehydration. The difference was statistically significant (p = 0.005, see Figure 2).

## Discussion

CAP represents a common cause of hospital admission for children. Recent evidence based guidelines for management, investigation and treatment for CAP in pediatric patients have been established, including admission criteria [2,6]. Many reports on CAP management in children have been published in the literature, but only a few on evaluation of hospital admission. In 2007, Clark et al. [5] evaluated hospital admission rates for childhood pneumonia from 13 Hospitals of United Kingdom. From 750 children assessed in hospital, incidence of CAP was 14.4/10,000 children per year and 33.8 for < 5-year-olds; with an incidence for admission to hospital of 12.2 and 28.7 respectively. Risk of severe CAP was significantly increased for those children aged < 5 years and with prematurity. It also varied significantly by county of residence. In the present report, we scored 135 pediatric patients observed at E.D. in 2 winter seasons because of CAP, whose 120 were admitted (88.8%). Furthermore, CAP incidence among admitted patients was 108/10,000 children for the analyzed period. Admission rate to our Unit for CAP resulted extremely high if related to total admissions rate for other diseases for the same period (88% vs. 25%): this is probably because CAP is considered a significant disease by people accessing E.D., who often did not consult their primary pediatrician before E.D. access.

We further assessed appropriate admission for CAP by performing a retrospective analysis on patients of a Pediatric Unit of a main General Hospital from Southern Italy. Evaluation of clinical criteria for correct admission allowed to score the presence of at least a single criterion (namely dyspnoea) in 43.3% of patients admitted (52 out of 120). As expected, infants presented with dyspnoea in a higher proportion (62.2%). All patients presenting with low oxygen saturation also had dyspnoea. Dyspnoea is variously defined as unpleasant or uncomfortable breathing, usually by adult pneumologists [6]. Dyspnoea assessment by subjective parameters is very difficult among pediatric patients as their evaluation is not usually possible especially among infants and younger babies, and appears difficult in children; on the other hands, objective parameters (respiratory rate, chest retractions, etc.) can be easily determined, thus reflecting clinical severity status. Even if evaluation of oxygen saturation by pulse oximetry represents an easy and careful method to estimate respiratory impairment, it could, if normal, underestimate the need of hospital admission. Thus, from our data it appears that dyspnoea represents the main clinical criterion for appropriate CAP children admission.

Inflammatory indices (ESR and CRP) were found elevated in 33.3% of patients. Analysis of combined parameters revealed that values of at least a single parameter were found raised in preschool and school children more than in infants. Positivity for anti-Mycoplasma pneumoniae antibodies was scored in 20.4% of patients, less frequently among infants (2.2%) than among preschool (22.9%), and school children (21.4%). Evaluation of data on inflammatory indices and etiology mostly reflects age specific agents for CAP.

As to poor feeding/dehydration, most patients requiring i.v. fluids supplementation presented with dyspnoea ± low SaO_2_. This finding appears reasonable as more serious respiratory impairment usually leads to dehydration and/or poor feeding, requiring fluids supplementation. Dehydration was equally found among infants and older children: even if this finding seems atypical, it is to consider that infants requiring i.v. fluids are usually more severely affected, often needing intensive respiratory care, thus possibly not included in this study.

Ability of family to provide appropriate observation or supervision was specifically assessed by evaluating their social status appraised by geographic area origin. We found that 76% of admitted patients derived from socially depressed areas of Naples and surroundings. A higher frequency of E.D. accessing (and consequently hospitalizing) among patients from socially depressed areas was found (see Figure 1). In 2000, Glazier et al. [9] evaluated relationship between socioeconomic factors and hospital use in Canadian context. Population-based rates of admission to hospital, bed days and costs were all significantly related to census tract income. They concluded that poor urban neighborhoods may require more resources, related to higher hospital admission and readmission rates. In 2003, Barnett et al. [10] examined the relationship between deprivation and changing patterns of public hospital admissions in 1992-1997 period, in New Zealand. They revealed that differences between admission rates for people living in the most and least deprived areas increased over time, especially following the implementation of the 1993 health reforms. Furthermore, patients from more affluent areas were hospitalized longer than low-income patients. They also suggested that the widening social gap in hospitalization rates is a result of the effects of poverty and problems of access to primary care.

Finally, presence of dyspnoea and/or SaO2 ≤ 92%, and/or dehydration was scored only in about 35% of "at social risk" patients, in contrast with 66% of "not at social risk" patients (Figure 2): these data confirm that in social deprived population clinical severity is not the main cause for hospital assessment request and admission.

Our findings represent an important issue reproducing more general data on deprivation status of Southern Italy, particularly of Naples and its surrounding areas, in comparison with other Italian regions [8]. We disclosed that social deprivation status represents the main criterion for hospital admission in our geographic area.

## Conclusion

In our study we found that presence of dyspnoea represents the main clinical criterion for appropriate children admission for CAP. This criterion is quite easy to evaluate and results extremely useful especially in case of lacking other more objective parameters (i.e. oxygen pulse oximetry), or when patients discomfort is more severe then indicated by trans-cutaneous SaO2 levels.

On the other hand, family inability to children care reflecting social deprivation status represents the main overall criterion for hospital admission in our geographic area. Accurate evaluation of family ability to provide appropriate observation/supervision is strongly recommended in deciding for children hospital admission, especially in some social context where people often directly access E.D., because they by-pass primary pediatricians or because of some difficulty in consulting them. A stronger cooperation between hospital and primary care pediatricians could reduce direct E.D. patients access and their inappropriate admission. In this direction, some cooperation efforts have been made in some circumstances, e.g. by using common and shared protocols on specific clinical topics [11], or by directly involving primary care pediatricians in hospital management of admitted children. A further and more effective intervention would be the direct involvement of primary care pediatricians in H24 medical outpatients assistance, as recently stated by Italian Government [12]: this issue would really reduce direct access to E.D. and consequently inappropriate hospital admission.

## Competing interests

The authors declare that they have no competing interests.

## Authors' contributions

FA conceived the study and collected data. DDB analyzed results and drafted the manuscript. PS conceived the study and polished the manuscript.

All authors read and approved the final manuscript.
